# Effects of the Feeding Frequency of Frozen Housefly Larvae on the Growth Performance, Physiological Functions, and Intestinal Microbiota of *Cherax quadricarinatus*

**DOI:** 10.1155/anu/9988505

**Published:** 2026-08-20

**Authors:** Zhuang Mai, Jie Wei, Yakun Wang, Sikai Xu, Qiaoyan Zhou, Tianhui Jiao, Lingyun Yu

**Affiliations:** ^1^ Key Laboratory of Tropical and Subtropical Fishery Resource Application and Cultivation of Ministry of Agriculture and Rural Affairs, Pearl River Fisheries Research Institute, Chinese Academy of Fishery Sciences, Guangzhou 510380, China, cafs.ac.cn

**Keywords:** *Cherax quadricarinatus*, growth performance, immunity-related gene, intestinal microbiota, *Musca domestica*

## Abstract

Optimal feeding strategies for the red claw crayfish, *Cherax quadricarinatus*, remain to be established. In this study, *C. quadricarinatus* was fed frozen housefly larva meal (*Musca domestica* [MD]) at different frequencies to evaluate effects on growth performance, antioxidant capacity, digestive enzyme activity, immune genes, and intestinal microbiota structure. Four feeding protocols were established: control (C) fed basic feed, experimental group 1 (E1) fed basic feed for 2 days and then compound feed (50% MD + 50% basic feed) for 1 day, experimental group 2 (E2) fed basic feed for 1 day and then compound feed for 1 day, and experimental group 3 (E3) fed compound feed daily. After 10 weeks, the weight gain rate (WGR) was significantly higher in E3 than in the other three groups (*p* < 0.05), while the survival rate (SR), specific growth rate (SGR), and hepatosomatic index (HSI) did not differ among groups (*p* > 0.05). In terms of physiological processes, lipase (LPS) and trypsin (TPS) activities were higher, expression levels of the immune‐related gene lysozyme (*LZM*) were significantly higher, and acid phosphatase (ACP) activity was significantly higher in E2 than in the control group (*p* < 0.05). E2 showed the highest ecdysone receptor (*EcR*) and retinoid X receptor (*RXR*) expression levels, related to molting regulation. In the intestinal microbiota, there were no differences in α‐diversity indices among groups; however, E1 increased the abundance of the phylum Bacteroidota. Consequently, while daily MD feeding (E3) maximized weight gain, intermittent feeding (E2) offered a superior balance between growth performance and physiological homeostasis, including digestion, immunity, and molting regulation. These findings provide a theoretical basis for precision farming of *C. quadricarinatus*.

## 1. Introduction

With the continuous growth of the global population and rising demand for high‐quality animal protein, the aquaculture industry has become a critical sector for ensuring food security owing to the high protein conversion efficiency. Data from the Food and Agriculture Organization of the United Nations [1] indicate that global aquaculture production has increased steadily. In this context, developing methods to improve breeding efficiency and product quality has become a core aim for industry development. Nutritional regulation, as a key process throughout the aquaculture cycle, directly determines the growth performance, health status, and economic benefits of cultured organisms, making it a major focus in both scientific research and industrial practices. In aquaculture operations, the rational application of nutritional supplements can compensate for nutritional deficiencies in basal feeds and optimize physiological processes via functional components [2]. However, traditional supplementary feeds, such as fresh or frozen wild fish, despite their nutritional density, pose significant challenges regarding water eutrophication, water quality management, and the risk of pathogenic bacterial contamination [3]. Consequently, developing environmentally friendly, efficient, and safe supplements has become an urgent goal for promoting the green and sustainable development of the aquaculture industry.

In recent years, insect resources have demonstrated significant application potential in aquafeeds owing to their high nutritional value, short life cycle, and strong production sustainability [4, 5]. In particular, housefly (*Musca domestica [MD]*) larvae (fly maggots), characterized by a high protein content (60.88%), balanced amino acid composition (meeting WHO/FAO standards), and abundant bioactive substances (such as antimicrobial peptides and lectins), are regarded as effective natural immune enhancers [6, 7]. Fly maggots can significantly improve the growth rate of Nile tilapia (*Oreochromis niloticus*) and African sharptooth catfish (*Clarias gariepinus*) [8, 9], enhance the antioxidant capacity of yellow catfish (*Pelteobagrus fulvidraco*) [10], and optimize the intestinal microbiota structure of swamp eel (*Monopterus albus*) [11]. Their functional diversity provides a solid theoretical and practical foundation for replacing traditional supplementary feed.

Red claw crawfish (*Cherax quadricarinatus*), native to northern Australia, has become an economically important freshwater aquaculture species owing to its delicious meat and high meat yield [12, 13]. However, nutritional research has predominantly focused on protein and lipid ratios in basal feeds [14, 15] and the effects of trace additives [16]. However, systematic evaluations of functional supplementary feeds remain scarce. Despite evidence for the beneficial effects of fly maggots on other aquatic taxa, their effects on the growth performance, immune regulation, and intestinal health of *C. quadricarinatus* are not clear. In particular, the differential impacts of various feeding frequencies have not been determined. This study clarifies the applicability and optimal feeding frequency of fly maggots as nutritional supplements based on the growth performance, antioxidant capacity, digestive enzyme activity, immune gene expression, and intestinal microbiota structure of *C. quadricarinatus*. The findings provide a theoretical foundation for precision farming of *C. quadricarinatus* while promoting the efficient and sustainable utilization of insect resources in aquafeed to support the green transition of the industry.

## 2. Materials and Methods

### 2.1. Experimental Diets and Design

The basal diet used in the experiment was purchased from Charoen Pokphand Aquatic Co., Ltd. (Yangjiang, Guangdong, China). This feed was scientifically formulated with high‐quality raw materials, including fish meal, soybean meal, wheat flour, and fish oil. Its crude protein, crude fat, crude ash, and moisture contents were 35.0%, 4.0%, 16.0%, and 12.0%, respectively, all meeting relevant standards for aquatic animal feed. The fly maggots used in the experiment were supplied by Hengzhao Lanlong (Guangdong) Aquatic Products Co., Ltd. (Jiangmen, Guangdong, China), and their nutritional composition is shown in Table 1 [17]. These maggots underwent strict screening and quality testing to ensure compliance with experimental requirements and were stored in a −20°C cold storage facility. Before feeding, the maggots were thawed for 30 min and then mixed with feed for administration. To avoid uneven feed intake among individuals due to *C. quadricarinatus* competition and physiological issues caused by excessive single‐dose protein intake, the daily maggot feeding amount was controlled at 50% of the total daily intake. Specifically, 50% frozen MD was mixed with 50% basal diet to form the supplemented diet. The experiment included four treatment groups: control group (C) fed the basal diet throughout as the experimental reference baseline; experimental group 1 (E1) fed basal feed for 2 days followed by 1 day of feeding with mixed feed (50% MD mixed with 50% basal diet); experimental group 2 (E2): with 1 day of the basal diet combined with 1 day of the mixed feed (also 50% MD + 50% basal diet); and experimental group 3 (E3): fed the mixed feed throughout (50% MD + 50% basal diet). The experiment lasted 10 weeks to ensure sufficient time for observing and recording all changes.

**Table 1:** Nutrient composition of *Musca domestica* [17].

Formulation	Content
Amino acids (mg/100 g)	
Ala	4.85
Asp	2.21
Thr	2.77
Ser	5.63
Glu	5.71
Pro	1.58
Gly	3.27
Val	2.92
Met	2.34
Ile	1.46
Leu	2.90
Tyr	3.17
Phe	4.55
Lys	5.22
His	1.98
Arg	3.63
Total amino acids	5.80
Fatty acid (% of total fatty acids)	
C14:0	6.83
C16:0	26.74
C16:1n7	25.92
C18:0	2.32
C18:1n9c	21.75
C18:2n6c	16.44
SFA	35.89
UFA	64.11
Proximate composition (%)	
Protein	63.99
Fat	2.40

### 2.2. Experimental Animals and Operation

We followed the methods described in the authors’ previous study [18]. Juvenile *C. quadricarinatus* were also sourced from Hengzhao Lanlong (Guangdong) Aquatic Products Co., Ltd. (Jiangmen, Guangdong, China), with selection ensuring healthy, disease‐free, and vigorous individuals. Prior to the experiment, *C. quadricarinatus* were transferred to acclimation tanks for a 2‐week conditioning period. During this phase, the experimental aquaculture environment was simulated, and a small amount of basal diet was provided to enable shrimp to gradually adapt to trial conditions, minimizing interference from environmental changes. Postacclimation, 720 juvenile *C. quadricarinatus* with a uniform size (initial body weight: 7.41 ± 0.56 g) were selected from the population and randomly allocated to 16 glass tanks (150 cm × 58 cm × 31 cm) using a randomized grouping method, with 45 shrimp per tank, divided into four groups with three replicates each. During the experiment, feeding was conducted twice daily at 8:00 and 17:00, with the feeding amount calculated as 4% of the total shrimp body weight and adjusted appropriately based on feeding behavior and growth stage. Residual feces and uneaten feed were promptly removed via siphoning 1–2 h postfeeding to maintain clean aquaculture water and prevent water quality deterioration. Throughout the culture period, key water quality parameters including temperature, dissolved oxygen (DO), pH, and ammonia nitrogen were regularly monitored using an Octadem W‐II water quality analyzer (Octadem Technology, Inc., Wuxi, China). Water temperature was maintained at 25–28°C, DO above 6 mg/L, pH stabilized at 7.5–8.5, and ammonia nitrogen below 0.1 mg/L, ensuring stable conditions optimal for *C. quadricarinatus* growth.

### 2.3. Sample Collection and Determination

Samples were collected in strict accordance with the guidelines of the animal experimental ethics review board of the Pearl River Fisheries Research Institute, Chinese Academy of Fishery Sciences (LAEC‐PRFRI‐2023‐10‐03), fully respecting laboratory animal welfare. At the end of the 10‐week culture experiment, shrimp were starved for 24 h to empty the gastrointestinal tract, minimizing the effects of food residues on subsequent measurements. Subsequently, crayfish in each tank were precisely counted to calculate the survival rate (SR). A Mettler Toledo AL‐204 precision balance (Mettler Toledo, Inc., Shanghai, China) was used to accurately measure the body weight and hepatopancreas wet weight of crayfish from each group. Fifteen shrimp per group were randomly selected for hepatopancreas tissue collection to assess the enzyme activity and gene expression levels; intestinal tissues from the remaining shrimp in each group were collected for analyses of the intestinal microbiota. All collected tissue samples were immediately flash‐frozen in liquid nitrogen to maximally preserve intracellular biomolecule activity and were then transferred to a −80°C freezer for long‐term storage. Growth indices were calculated using methods proposed by Liu et al. as follows [19]:

Survival rate SR,%=Final crayfish number/Initial crayfish number×100;

Weight gain rate WGR,%=Final body weight−Initial body weight/Initial body weight×100;

Specific growth rate SGR,% day−1=ln Final body weight−ln Initial body weight/Days×100;

Hepatosomatic index HSI,%=Hepatopancreas wet weight/Final body weight×100.

### 2.4. Biochemical Analysis

Hepatopancreas samples from crayfish were collected to determine antioxidant, immune, and digestive enzyme activity levels. Superoxide dismutase (SOD) activity was measured using the WST‐1 method. Catalase (CAT) activity was determined using the ammonium molybdate method, with enzyme activity calculated based on absorbance. Glutathione peroxidase (GSH‐Px) activity was assayed calorimetrically by detecting changes in reaction substrates during the GSH‐Px–catalyzed reaction between glutathione and hydrogen peroxide. Acid phosphatase (ACP) activity was measured using a microplate enzymatic assay. Trypsin (TPS) and lipase (LPS) activities were determined using microplate assays. All enzyme activities were analyzed using kits produced by Nanjing Jiancheng Bioengineering Institute (Nanjing Jiancheng Bioengineering Institute, Nanjing, China), with sample pretreatment, reagent preparation, and measurements strictly following kit instructions to ensure the accuracy and reliability of experimental results.

### 2.5. Immune‐ and Molting‐Related Gene Expression

Total RNA was isolated from hepatopancreas samples using the TRIzol Reagent Kit (Life Technologies, Carlsbad, CA, USA) following standard protocols to extract high‐quality RNA. The concentration and purity of extracted RNA were quantified through spectrophotometry (OD260/OD280) using a NanoDrop8000 spectrophotometer (Thermo Scientific, Waltham, MA, USA). Integrity and quality were analyzed by 1.0% agarose gel electrophoresis to observe potential RNA degradation bands. Total RNA was reverse‐transcribed into cDNA using the M‐MLV Reverse Transcriptase Kit (Invitrogen, Waltham, MA, USA) according to the manufacturer’s instructions. All RNA and cDNA samples were stored in a −80°C freezer. The expression levels of two immune‐related genes (signal transducer and activator of transcription [*STAT*] and lysozyme [*LZM*]) commonly studied in crustaceans and two molting‐related genes (ecdysone receptor [*EcR*] and retinoid X receptor [*RXR*]) were evaluated. The primer sequences for target and reference genes are listed in Table 2. Primers were designed based on conserved sequences in crayfish, ensuring specificity and amplification efficiency. Real‐time fluorescent quantitative PCR (qPCR) was performed using a Step One Plus Real‐time PCR system (Applied Biosystems, Foster City, CA, USA). The reaction mixture contained 1 μL (50 ng/μL) of cDNA, 10 μL of 2×SYBR Green Premix (Universal), 1 μL (10 pmol/μL) of each primer, and 7 μL of ddH_2_O, with a final volume of 20 μL. The qPCR protocol was as follows: 95°C predenaturation for 30 s; 35 cycles of 95°C denaturation for 40 s, 60°C annealing for 45 s, and 72°C extension for 30 s; followed by a final 72°C extension for 10 min. Gene expression levels were calculated using the 2^−ΔΔCt^ method.

**Table 2:** The primer sequences used in the real‐time quantitative PCR.

Primer name	Primer sequences (5′–3′)	Efficiency values (%)	Sources
*β*‐*Actin*‐F	CCCCATGCTATCTTGCGTCT	98.92	MN396754.1
*β*‐*Actin*‐R	CGTCAGGAAGCTCGTAGGAT		
*STAT* ‐F	CTTCGCTATCCGTCCTCT	90.05	MT103423.1
*STAT* ‐R	AATGCTTGTTCCTTGGGT		
*LZM*‐F	GCAACAGGAACGGTAGCAAG	90.32	Lu et al., 2019
*LZM*‐R	GCCACCCAAGCGGAATAT		
*EcR*‐F	GGTTTCGGCACTCTTCAACG	92.35	OL963596.1
*EcR*‐R	ACAGATTGCGACAAAAGCGG		
*RXR*‐F	AGGAGATGCCGTAACCAACA	99.23	KM016907.1
*RXR*‐R	ATGCTTCGGTGTGAGAAGGA		

### 2.6. 16S rRNA Gene Sequencing

Intestinal samples from each group of *C. quadricarinatus* were collected for microbial diversity analysis. Following the methods of Liu et al., DNA was extracted from samples using a kit (Guangzhou Meiji Biotechnology Co., Ltd.), and DNA quality was detected using a spectrophotometer (Thermo Fisher Scientific, Shanghai, China). For qualified DNA samples, the V3 + V4 regions of 16S rDNA were amplified using barcode‐specific primers. The PCR reaction mixture had a total volume of 30 µL (1.5 µL of template DNA, 3.0 µL of 10× Ex Taq Buffer, 2.4 µL of dNTP Mix [2.5 mM each], 0.6 µL each of forward and reverse primers [10 µM], 0.15 µL of TaKaRa Ex Taq [Takara Bio, Japan], and 21.75 µL of nuclease‐free water). Selected primer sequences were as follows: 341F: CCTACGGGNGGCWGCAG and 806R: GGACTACHVGGGTATCTAAT. After the first round of PCR amplification, the products were purified using AMPure XP Beads and quantified with Qubit 3.0. A second round of amplification was performed, and the resulting products were again purified with AMPure XP Beads. PCR was run for 28–35 cycles, and the amplicons were verified by agarose gel electrophoresis. Subsequently, sequencing libraries were constructed and sequenced on a MiSeq platform (Illumina, USA).

Sequence data in the FASTQ format were processed on the OmicShare platform (https://www.omicshare.com/). Reads were denoised and filtered using the DADA2 plugin, and sequences were classified based on the SILVA 132 database. Taxonomic classification at the phylum and genus levels was visualized using stacked bar plots of relative abundance generated on the OmicShare platform (https://www.omicshare.com/). Alpha diversity was analyzed using the Chao1, Simpson, and Shannon indices, and statistical analysis was performed using GraphPad Prism 9.5 software (GraphPad, USA) [20].

### 2.7. Statistical Analysis

Experimental data are expressed as the mean ± standard error (mean ± SE) of three replicates. All statistical analyses were performed using GraphPad Prism 9.5 software. First, the Shapiro–Wilk test was applied to verify data normality across groups; Levene’s test was used to confirm the homogeneity of variances. If data satisfied both normality and homoscedasticity assumptions, one‐way ANOVA was employed for intergroup comparisons, with Tukey’s test for post hoc multiple comparisons. If variances were unequal, Welch’s ANOVA was applied. For data violating normality or homogeneity of variances, the nonparametric Kruskal–Wallis test was utilized. Statistical significance was defined at *p* < 0.05.

## 3. Results

### 3.1. Growth Performance

An in‐depth evaluation of growth performance in *C. quadricarinatus* across different treatment groups was conducted, with detailed data presented in Table 3. No significant differences in SR were observed among the groups (*p* > 0.05), with values in the control group (C) and experimental groups E1, E2, and E3 of 88.33 ± 3.20%, 85.00 ± 6.89%, 85.00 ± 6.31%, and 93.33 ± 2.72%, respectively. Weight gain rate (WGR) ranged from 162.23 ± 7.62% to 218.52 ± 14.21%. Notably, E3 exhibited the highest WGR at 218.52 ± 14.21%, which was significantly higher than those of C (162.23 ± 7.62%) and E2 (167.82 ± 7.60%) (*p* < 0.05). However, no significant difference was observed between E3 and E1 (182.76 ± 7.36%) (*p* > 0.05). There were no significant differences in specific growth rate (SGR) and hepatosomatic index (HSI), critical indicators of growth performance, among groups (*p* > 0.05). The measured SGR ranged from 1.36 ± 0.04% day^−1^ to 1.50 ± 0.06% day^−1^, while the HSI values varied between 6.95 ± 0.29% and 7.78 ± 0.52%.

**Table 3:** Effects of *Musca domestica* supplementation on growth performance in *C. quadricarinatus*.

Parameters	Groups			
	C	E1	E2	E3
SR (%)	88.33 ± 3.20	85.00 ± 6.89	85.00 ± 6.31	93.33 ± 2.72
WGR (%)	162.23 ± 7.62^b^	182.76 ± 7.36^ab^	167.82 ± 7.60^b^	218.52 ± 14.21^a^
SGR (%day^−1^)	1.36 ± 0.04	1.44 ± 0.05	1.39 ± 0.05	1.50 ± 0.06
HSI (%)	6.95 ± 0.29	7.78 ± 0.52	7.18 ± 0.27	7.64 ± 0.40

*Note:* Data are reported as the mean ± SE of four replicates. Data with different letters were significantly different (*p* < 0.05) among all the treatments.

Abbreviations: HSI, hepatosomatic index; SGR, specific growth rate; SR, survival rate; WGR, weight gain rate.

### 3.2. Antioxidant Capacity and Immune and Digestive Enzyme Activities

**Figure 1:**
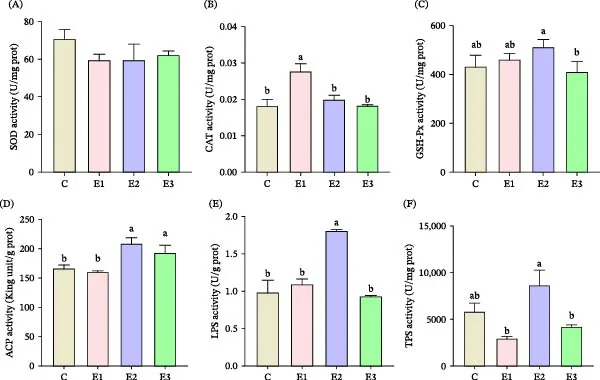
Effects of different MD feeding amounts on antioxidant and digestive enzyme activity in the hepatopancreas of *C. quadricarinatus*. (A) Superoxide dismutase (SOD), (B) peroxidase (CAT), (C) glutathione peroxidase (GSH‐Px), (D) acid phosphatase (ACP), (E) lipase (LPS), and (F) trypsin (TPS). Different letters above the error lines indicate significant differences (*p* < 0.05).

The effects of MD feeding on antioxidant, immune enzymes, and digestive activity in the hepatopancreas of *C. quadricarinatus* are shown in Figure 1A–F. Levels of SOD, a crucial antioxidant enzyme, ranged from 59.59 to 70.62 U/mg prot, with no significant intergroup differences (*p* > 0.05) (Figure 1A). CAT activity ranged from 0.018 to 0.028 U/mg prot. Notably, E1 showed the highest CAT activity at 0.028 ± 0.002 U/mg prot, which was significantly higher than levels in the other three groups (*p* < 0.05) (Figure 1B). GSH‐Px activity varied between 409.85 and 511.58 U/mg prot. E2 displayed the highest GSH‐Px activity (511.58 ± 25.30 U/mg prot), surpassing that in E3 significantly (*p* < 0.05) but not significantly different from those in other groups (Figure 1C). Regarding immune and digestive enzymes, ACP activity ranged from 160.21 to 208.92 King unit/g prot. Levels of ACP activity were significantly higher in E2 (208.92 ± 9.89 King unit/g prot) and E3 (192.80 ± 13.48 King unit/g prot) than in C (166.40 ± 5.76 King unit/g prot) and E1 (160.21 ± 1.96 King unit/g prot) (*p* < 0.05) (Figure 1D). LPS activity was highest in E2 at 1.81 ± 0.03 U/mg prot, exceeding those in C, E1, and E3 significantly (*p* < 0.05) (Figure 1E). TPS activity in E2 was significantly higher than those in E1 and E3 (*p* < 0.05) but did not differ significantly from that in C (*p* > 0.05) (Figure 1F).

### 3.3. Immune‐ and Molting‐Related Gene Expression

**Figure 2:**
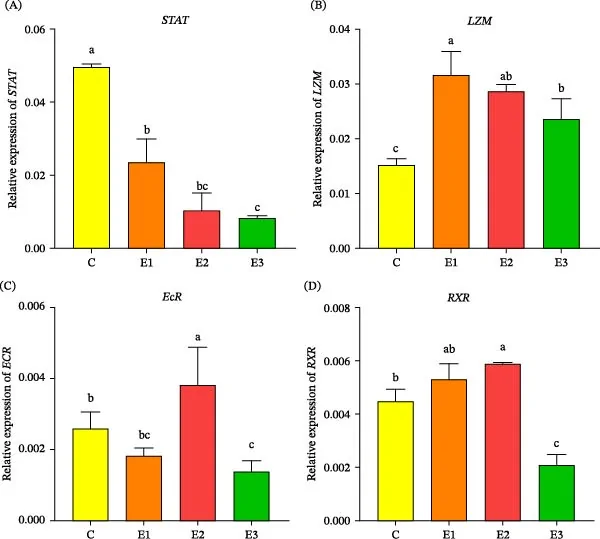
Effects of different MD feeding amounts on immunity and molting gene expression levels in the hepatopancreas of *C. quadricarinatus*. (A) signal transducer and activator of transcription (*STAT*), (B) lysozyme (*LZM*), (C) ecdysone receptor (*EcR*), and (D) retinoid X receptor (*RXR*). Different letters above the error lines indicate significant differences (*p* < 0.05).

The effects of different maggot‐feeding treatments on the expression of immune and molting‐related genes in the hepatopancreas of *C. quadricarinatus* are shown in Figure 2A–D. For nonspecific immune genes, as the maggot feeding amount increased, the expression level of the *STAT* gene decreased gradually. The *STAT* gene expression level in the control group (C) was significantly higher than those in E1 and E2 (*p* < 0.05) and extremely significantly higher than that in E3 (*p* < 0.01) (Figure 2A). In contrast, the *LZM* expression level increased significantly in the maggot‐fed groups. The *LZM* gene expression levels in all three experimental treatment groups were significantly higher than that in the control group (C) (*p* < 0.05) (Figure 2B). For the molting‐related genes, significant differences in the expression levels of *EcR* and *RXR* were found among treatment groups. *EcR* gene expression was higher in E2, significantly higher than those in C and E1 (*p* < 0.05) and extremely significantly higher than that in E3 (*p* < 0.01) (Figure 2C). The expression level of the *RXR* gene was also relatively high in E2, significantly higher than that in C (*p* < 0.05) and extremely significantly higher than that in E3 (*p* < 0.01) (Figure 2D).

### 3.4. Intestinal Microbiota Structure

**Figure 3:**
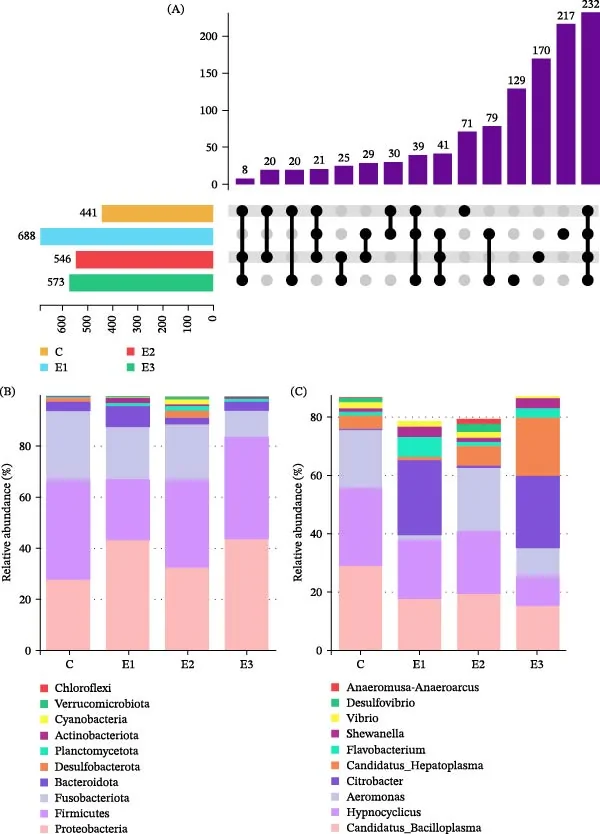
Effects of different MD feeding amounts on the intestinal microbiota of *C. quadricarinatus*. (A) UpSet plot comparing the relationships of intestinal microbiota OTUs in different groups. The left panel displays bars with colors corresponding to each treatment, representing the number of OTUs. Black circles in the matrix indicate sets of treatments that intersect. (B) Relative abundances of the top 10 intestinal bacteria at the phylum level. (C) Relative abundances of the top 10 intestinal bacteria at the genus level.

The results of the α‐diversity analysis of the gut microbiota in the four groups of *C. quadricarinatus* using nonparametric statistical methods are shown in Table 4. The sequencing coverage for each group exceeded 99%, indicating the reliability and validity of the diversity data obtained in this experiment. There were no significant differences among groups in terms of Chao1, Shannon, and Simpson indices (*p* > 0.05). Through in‐depth analyses using high‐throughput sequencing technology, 2833 OTUs were identified in the gut samples of the four groups of *C. quadricarinatus*. Among these, 232 OTUs were shared across all four groups, while there were 71, 217, 170, and 129 unique OTUs for C, E1, E2, and E3, respectively (Figure 3A). Multivariate statistical analysis of beta‐diversity revealed that E3 diverged more prominently from the other groups, while no significant difference was observed between E1 and E2 (Figure S1). Dominant phyla with relative abundances greater than 5% identified in all four groups included Proteobacteria (relative abundance ranging from 27.81% to 43.67%), Firmicutes (23.96% to 40.21%), and Fusobacteriota (10.19% to 26.87%), with no significant differences in the abundance of these dominant phyla among groups (*p* > 0.05). Notably, Bacteroidota was dominant only in E1, with a relative abundance of 6.2 ± 0.5% (Figure 3B). At the genus level, the dominant genera shared among the four groups were *Candidatus_Bacilloplasma* and *Hypnocyclicus*. The relative abundance of *Candidatus_Bacilloplasma* in C was significantly higher than those in the other three groups (*p* < 0.05), while the abundance of *Hypnocyclicus* in E3 was significantly lower than those in C, E1, and E2 (*p* < 0.05) (Figure 3C).

**Table 4:** Alpha diversity indices of intestinal microbiota of *C. quadricarinatus* after the 10‐week feeding trial.

Alpha diversity indices	Groups			
	C	E1	E2	E3
Chao1	518.71 ± 44.62	683.26 ± 77.81	536.27 ± 46.55	541.79 ± 100.77
Shannon	3.00 ± 0.57	3.39 ± 0.81	3.72 ± 0.67	3.48 ± 0.44
Simpson	0.72 ± 0.12	0.70 ± 0.16	0.81 ± 0.07	0.81 ± 0.03
Coverage (%)	99.88 ± 0.00	99.84 ± 0.00	99.89 ± 0.00	99.89 ± 0.00

*Note:* Data are reported as the mean ±SE of four replicates.

## 4. Discussion

In recent years, MD has emerged as a research hotspot in aquafeed owing to its high nutritional value, balanced essential amino acids, and sustainable production traits [6]. Maggots are rich in vitamins, trace elements, and functional peptides, which can enhance aquatic animal growth performance by promoting digestive enzyme secretion and improving immunity [21]. In this study, continuous maggot feeding (E3) increased the WGR of *C. quadricarinatus* significantly but did not affect SGR. This outcome highlights the “double‐edged” nature of high‐protein diets; although the high‐quality protein and balanced amino acids in maggots effectively meet the nutritional demands of crustaceans [22], excessive intake may induce metabolic burden on the hepatopancreas, characterized primarily by excessive lipid deposition [23]. This study found that the HSI in Group E3 was slightly higher than that in the control group, consistent with previous findings for *Macrobrachium rosenbergii*. This phenomenon may stem from metabolic overload in the hepatopancreas, leading to reduced digestive enzyme activity, causing nutrient retention in the hepatopancreas, and resulting in an increase in the weight of the dry hepatopancreas [22]. Notably, periodic feeding strategies (E2, fed every 2 days) enhanced digestive enzyme activities significantly (TPS and LPS), thereby optimizing nutrient absorption efficiency. The elevated TPS activity may be attributed to the activation of TPS secretion pathways by small peptides in maggots [24], while increased LPS activity is hypothesized to result from long‐chain fatty acids in maggots, which stimulate LPS activity [25]. This energy allocation strategy of metabolic compensatory mechanisms aligns closely with results for loach (*Misgurnus anguillicaudatus*), showing that 30% maggot in fish meal yields the optimal WGR [26], further revealing that crustaceans exhibit a nonlinear dose–response relationship to nutrient input; moderate feeding achieves a balance between growth and physiological functions through metabolic regulation, whereas sustained high‐intensity feeding may trigger metabolic adaptive fatigue.

In aquaculture, feeding strategies directly influence the physiological functions of cultured organisms [27]. In this study, there were significant “dose‐effect” differences in the antioxidant capacity of *C. quadricarinatus* under varying MD feeding frequencies. E1 (fed every 3 days) exhibited significantly higher CAT activity than those in other groups, while E2 showed significantly elevated GSH‐Px activity compared with that in E3, with no intergroup differences in SOD activity. This phenomenon relates to the hierarchical response mechanism of the antioxidant system. SOD, as a primary antioxidant enzyme, converts superoxide anions (O_2_^-^) into hydrogen peroxide (H_2_O_2_) [28]; CAT and GSH‐Px maintain redox homeostasis by scavenging H_2_O_2_ and lipid peroxides [29, 30]. The iron‐rich composition of MD enhances CAT and GSH‐Px activity by serving as enzymatic cofactors [31]. In contrast, continuous feeding in E3 reduced the antioxidant response efficiency due to feedback inhibition. Regarding immune function, ACP activity increased significantly in E2 and E3, which could be attributed to the immunomodulatory effects of antimicrobial peptides and lectins in MD [32]. Antimicrobial peptides exert bactericidal functions by disrupting pathogen membrane integrity [33], while lectins recognize pathogen glycosylation patterns, activating the prophenoloxidase (PO) system to enhance nonspecific immunity [34]. Elevated ACP activity further indicates heightened phagocyte activity, consistent with improved hemolymph immune parameters in Pacific white shrimp (*Litopenaeus vannamei*) fed MD‐supplemented diets [35]. Although SOD and GSH‐Px activities remained unchanged, intermittent feeding (E1 and E2) resulted in the synergistic regulation of oxidative stress and immune defense through optimized CAT and ACP activities. This feeding pattern aligns with the natural feeding rhythm of crustaceans, avoiding metabolic adaptive fatigue caused by sustained high‐intensity feeding. These results reinforce the potential of MD as functional feed additives; however, precise optimization of the feeding frequency and dosage is essential to balance growth promotion and stress resistance.

Crustaceans lack the adaptive immune system specific to vertebrates and rely heavily on nonspecific immune responses for resistance. LZM, a core effector molecule of the innate immune barrier, can specifically recognize and hydrolyze the β‐1,4 glycosidic bonds of bacterial cell wall peptidoglycan, thereby disrupting the structural integrity of pathogens [36]. The data from this study show that maggot feeding increases the *LZM* expression levels significantly in *C. quadricarinatus*. Both E2 (fed every 2 days) and E3 (fed daily) showed significant differences in *LZM* levels compared with that in the control group (*p* < 0.05), with a clear dose‐dependent effect (Figure 2B). This result is consistent with the observation by Cheng et al. [35] that maggot feeding enhances the LZM activity in the hemolymph of *L. vannamei*, further confirming that maggot feeding can induce efficient transcription of the *LZM* gene in *C. quadricarinatus*. The JAK/STAT signaling pathway plays dual roles in crustacean immune regulation, participating in tissue repair under physiological conditions and being exploited by pathogens for replication during infection [37]. For example, white spot syndrome virus (WSSV) induces *STAT* phosphorylation and activates viral gene expression, thereby enhancing its own replication [38]. In this study, with increasing maggot feeding amounts, the expression of the *STAT* gene in *C. quadricarinatus* decreased significantly (Figure 2A), suggesting that immunomodulatory components in the maggot may influence the *STAT* expression in *C. quadricarinatus*. However, due to the current lack of studies on the relationship between *STAT* and WSSV in redclaw crayfish, future research could further investigate the effects of housefly larvae on WSSV challenge tests *C. quadricarinatus* [39]. Molting, as an important physiological process in the growth and development of crustaceans, is precisely regulated by the molting hormone (20‐hydroxyecdysone, 20E) and its receptor complex (*EcR*/*RXR*) [40, 41]. The heterodimer formed by *EcR* and *RXR* undergoes a conformational change upon binding to 20E and translocases into the cell nucleus, thereby activating the transcription of downstream molting‐related genes [42, 43]. This study confirmed that an intermittent feeding strategy using E2 (fed every 2 days) increased the expression levels of *EcR* and *RXR* significantly in *C. quadricarinatus* (*p* < 0.05). We hypothesize that intermittent nutrient intake mimics natural feeding rhythms, enhances the sensitivity of the *EcR/RXR* receptor complex to 20E, and thereby optimizes the molting process. The specific regulatory mechanisms need to be verified.

The gut microbiota of aquatic animals, acting as a key intermediary between the host and external environment, has substantial effects on host health and growth performance through various mechanisms, such as metabolic exchange, immune signaling regulation, and pathogen antagonism [44, 45]. In this study, using 16S rRNA sequencing technology, we analyzed various aspects of the gut microbiota of *C. quadricarinatus*, including species diversity. The results showed no significant differences in α‐diversity indices of the gut microbiota among the four groups, indicating that maggot feeding did not affect species richness and evenness of the gut microbiota in *C. quadricarinatus* significantly. This result is consistent with the strong ecological resilience of the gut microbiota in aquatic animals [46]. At the phylum level, Firmicutes, Proteobacteria, and Fusobacteriota constituted the core gut taxa and showed complex responses to different feeding strategies. As a representative of beneficial gut bacteria, Firmicutes produce metabolites that enhance the host’s digestive capacity and strengthen the tight junctions of intestinal epithelial cells, improving the physical barrier function of the gut substantially [20, 47]. Some bacteria in Proteobacteria, such as *Vibrio*, carry potential virulence factors. However, LPS in maggots can activate the TLR4/NF‐κB signaling pathway, moderately stimulating the host’s innate immunity and maintaining an immune balance [48]. Fusobacteriota are conditional pathogens, and their abundance is closely related to intestinal inflammatory responses [49]. In this study, the abundance of Fusobacteriota remained stable, and it is speculated that the antimicrobial peptides rich in maggots may inhibit the adhesion and proliferation of pathogens, effectively reducing the risk of gut infections. Additionally, in E1, the abundance of Bacteroidota increased significantly. This phylum can promote host nutrient absorption by degrading complex carbohydrates [50]. At the genus level, *Candidatus Bacilloplasma* and *Hypnocyclicus* were the dominant genera shared among all four groups. However, research on these genera is relatively limited, and their physiological effects on aquatic animals are not yet well understood. Future studies could combine bacterial strain isolation and culture with metabolomics analyses to further elucidate the physiological functions of these genera and clarify their interactions with host metabolism and immune defense mechanisms.

## 5. Conclusion

This study demonstrated the differential effects of different MD feeding strategies on the growth performance, physiological functions, and gut microbiota of *C. quadricarinatus*. In terms of growth performance, continuous maggot feeding (E3) increased the WGR of *C. quadricarinatus* significantly, demonstrating the growth‐promoting effect of maggots as a high‐protein feed. However, from a comprehensive physiological perspective, feeding maggots every 2 days (E2) was superior; E2 exhibited significantly enhanced digestive enzyme activities (LPS and TPS), increased expression of immune‐related genes (*LZM*), significantly increased ACP activity, and the highest expression levels of molting‐related genes (*EcR* and *RXR*). This group showed clear advantages in maintaining the balance of antioxidant, immune, and molting physiological functions. Feeding maggots every 3 days (E1) did not achieve the same WGR as that for E3 but increased antioxidant enzyme activity (CAT) and resulted in the unique presence of the phylum Bacteroidota, which improved the gut microbiota structure of *C. quadricarinatus* to some extent. In practical aquaculture, the appropriate maggot‐feeding strategy should be selected based on breeding objectives. Therefore, this study provides theoretical support for the refined breeding of *C. quadricarinatus* and offers important references for the efficient utilization of insect resources in the aquaculture feed sector and the sustainable development of the aquaculture industry.

## Author Contributions

**Zhuang Mai**: investigation, methodology, data curation, sampling, writing – original draft, writing – review and editing. **Jie Wei**: conceptualization, funding acquisition, writing – review and editing. **Lingyun Yu**: conceptualization, project administration, resources, writing – review and editing. **Yakun Wang**: conceptualization, project administration. **Qiaoyan Zhou and Tianhui Jiao**: formal analysis. **Sikai Xu**: sampling.

## Funding

This research was funded by the Central Public‐interest Scientific Institution Basal Research Fund, CAFS (Grants 2025SJGG1 and 2024SJRC1), the Guangdong Province Modern Agricultural Industrial Technology System Innovation Team Construction Project (Grant 2024CXTD25), and the China‐ASEAN Maritime Cooperation Fund (Grant CAMC‐2018F).

## Conflicts of Interest

The authors declare no conflicts of interest.

## Supporting Information

Additional supporting information can be found online in the Supporting Information section.

## Supporting information


**Supporting Information** Figure S1: Beta‐diversity analysis by PCoA.

## Data Availability

The data that support the findings of this study are available from the corresponding author upon reasonable request.
